# Rapid exome sequencing as a first-tier test in neonates with suspected genetic disorder: results of a prospective multicenter clinical utility study in the Netherlands

**DOI:** 10.1007/s00431-023-04909-1

**Published:** 2023-03-31

**Authors:** Richelle A. C. M. Olde Keizer, Abderrahim Marouane, Wilhelmina S. Kerstjens-Frederikse, A. Chantal Deden, Klaske D. Lichtenbelt, Tinneke Jonckers, Marieke Vervoorn, Maaike Vreeburg, Lidewij Henneman, Linda S. de Vries, Richard J. Sinke, Rolph Pfundt, Servi J. C. Stevens, Peter Andriessen, Richard A. van Lingen, Marcel Nelen, Hans Scheffer, Daphne Stemkens, Cor Oosterwijk, Hans Kristian Ploos van Amstel, Willem P. de Boode, Wendy A. G. van Zelst-Stams, Geert W. J. Frederix, Lisenka E. L. M. Vissers, on behalf of RADICON-NL consortium, L Henneman, M M van Haelst, E A Sistermans, M C Cornel, M Misra-Isrie, M M A M Mannens, Q Waisfisz, J M van Hagen, A S Brooks, T S Barakat, E H Hoefsloot, R A van Lingen, C A L Ruivenkamp, A van Haeringen, S Koene, G W E Santen, J W Rutten, B de Koning, S J C Stevens, A van den Wijngaard, M Sinnema, A P A Stegmann, M Vreeburg, M Vervoorn, P Andriessen, D Kasteel, E M Adang, A C Deden, H G Brunner, W P de Boode, H G Yntema, H Scheffer, W van Zelst-Stams, R Pfundt, T Kleefstra, A Marouane, L E L M Vissers, T Rigter, W Rodenburg, M A Swertz, V V AM Knoers, W S Kerstjens-Frederikse, R J Sinke, K J van der Velde, I M van Langen, M E van Gijn, J P van Tintelen, L S de Vries, G W J Frederix, J K Ploos van Amstel, K D Lichtenbelt, R A C M Olde Keizer, R Oegema, C Oosterwijk, D Stemkens

**Affiliations:** 1 Julius Center for Health Sciences and Primary Care, University Medical Center Utrecht, Utrecht University, Utrecht, Netherlands; 2 Department of Human Genetics, Radboud University Medical Center, Radboud Institute for Health Sciences, Nijmegen, Netherlands; 3 Department of Genetics, University Medical Center, University of Groningen, Groningen, Netherlands; 4 Department of Genetics, Utrecht University Medical Center, Utrecht, Netherlands; 5 Department of Pediatrics and Neonatology, Máxima Medical Center, Veldhoven, Netherlands; 6 Department of Clinical Genetics, Maastricht University Medical Center, Maastricht, Netherlands; 7 Department of Human Genetics and Amsterdam Reproduction and Development Research Institute, Amsterdam UMC, Vrije Universiteit, Amsterdam, Netherlands; 8 Department of Neonatology, University Medical Center Utrecht, Utrecht, Netherlands; 9 Department of Pediatrics, Máxima Medical Center, Veldhoven, Netherlands; 10 Department of Applied Physics, Eindhoven University of Technology, Eindhoven, Netherlands; 11 Department of Neonatology, Isala, Zwolle, Netherlands; 12 VSOP - National Patient Alliance for Rare and Genetic Diseases, Soest, Netherlands; 13 Department of Neonatology, Radboud University Medical Center, Radboud Institute for Health Sciences, Amalia Children’s Hospital, Nijmegen, Netherlands; 14 Department of Human Genetics, Donders Institute for Brain, Cognition and Behaviour, Radboud University Medical Center, Nijmegen, Netherlands

**Keywords:** Diagnostic workflow, Neonates, Economic evaluation, Clinical utility, Rapid exome sequencing

## Abstract

The introduction of rapid exome sequencing (rES) for critically ill neonates admitted to the neonatal intensive care unit has made it possible to impact clinical decision-making. Unbiased prospective studies to quantify the impact of rES over routine genetic testing are, however, scarce. We performed a clinical utility study to compare rES to conventional genetic diagnostic workup for critically ill neonates with suspected genetic disorders. In a multicenter prospective parallel cohort study involving five Dutch NICUs, we performed rES in parallel to routine genetic testing for 60 neonates with a suspected genetic disorder and monitored diagnostic yield and the time to diagnosis. To assess the economic impact of rES, healthcare resource use was collected for all neonates. rES detected more conclusive genetic diagnoses than routine genetic testing (20% vs. 10%, respectively), in a significantly shorter time to diagnosis (15 days (95% CI 10–20) vs. 59 days (95% CI 23–98, *p* < 0.001)). Moreover, rES reduced genetic diagnostic costs by 1.5% (€85 per neonate).

*Conclusion*: Our findings demonstrate the clinical utility of rES for critically ill neonates based on increased diagnostic yield, shorter time to diagnosis, and net healthcare savings. Our observations warrant the widespread implementation of rES as first-tier genetic test in critically ill neonates with disorders of suspected genetic origin.

**Table:** 

**What is Known:** *• Rapid exome sequencing (rES) enables diagnosing rare genetic disorders in a fast and reliable manner, but retrospective studies with neonates admitted to the neonatal intensive care unit (NICU) indicated that genetic disorders are likely underdiagnosed as rES is not routinely used.* *• Scenario modeling for implementation of rES for neonates with presumed genetic disorders indicated an expected increase in costs associated with genetic testing.*	
**What is New:** *• This unique prospective national clinical utility study of rES in a NICU setting shows that rES obtained more and faster diagnoses than conventional genetic tests.* *• Implementation of rES as replacement for all other genetic tests does not increase healthcare costs but in fact leads to a reduction in healthcare costs.*

**Supplementary Information:**

The online version contains supplementary material available at 10.1007/s00431-023-04909-1.

## Introduction

Genetic disorders are frequent causes of neonatal morbidity and mortality, and disease presentations are often undifferentiated at birth [1]. Since genetic disorders can progress fast, a rapid genetic diagnosis might provide the opportunity to reduce suffering, morbidity, and mortality, especially in critically ill infants and neonates [2, 3]. An essential indicator in genetic diagnostics is the turnaround time (TAT). Earlier research has demonstrated that rapid exome sequencing (rES) is related to a shortened TAT compared to conventional genetic diagnostic tests. The return of test results has decreased from several weeks or even months to a few days, thereby shortening the diagnostic odyssey and enabling precision medicine [4]. Genetic disorders and congenital anomalies (CA) affect around 6% of live births and are the leading reason for hospitalization in infants and neonates [5]. The presence of a genetic disorder can easily be missed because of the variable clinical presentation, often leading to a diagnostic odyssey requiring extensive evaluations, both clinically and genetically [6].

Approximately 2.5% of newborns is admitted to neonatal intensive care units (NICU) in the Netherlands [7]. The prevalence of genetic disorders is relatively high in critically ill newborns, and this is accompanied by long hospitalization and high healthcare utilization [8]. Genetic testing of newborns using rES at an earlier stage may reduce their diagnostic odyssey and enhance diagnosis-predicated precision. Early diagnosis may improve patient’s clinical outcome and can potentially be life-saving [2]. Identifying a genetic diagnosis can also help to avoid ineffective (intensive) care in critically ill newborns with poor prognosis.

Besides clinical implications, implementation of improved diagnostic tests can also have health economic impact. Gonzaludo et al. [9] have shown that children who are admitted to the hospital and diagnosed with a genetic disorder have a significant and disproportionate impact on resource use and related costs. By improving the diagnostic trajectory and shortening its length, future healthcare costs can be prevented, for example, by initiating adequate treatment earlier [10]. Before implementing a new technology, such as rES, into diagnostic care, it is important to understand the possible financial/economic consequences. Since the costs of performing rES have reduced over time, it is unknown what the actual costs will be when conventional genetic diagnostic trajectory is replaced by rES.

Based on several studies showing that rES provides a faster diagnosis, enabling timely precision medicine aiming to decrease morbidity and mortality of infants with genetic disorders [3, 5, 11–13], we hypothesized that rES can positively affect diagnostic yield and the length of the diagnostic trajectory at lower costs. However, rES is still not sufficiently implemented in clinical guidelines of critically ill neonates as standard genetic care. Our group recently indicated that a prospective follow-up study is needed, in which current genetic diagnostic costs are compared to a parallel diagnostic trajectory in which rES replaces all conventional genetic diagnostic testing [14]. Therefore, the aim of this study is to prospectively examine the clinical utility of rES versus conventional genetic testing, by comparing clinical and economic outcomes.

## Methods

### Study design

**Fig. 1:**
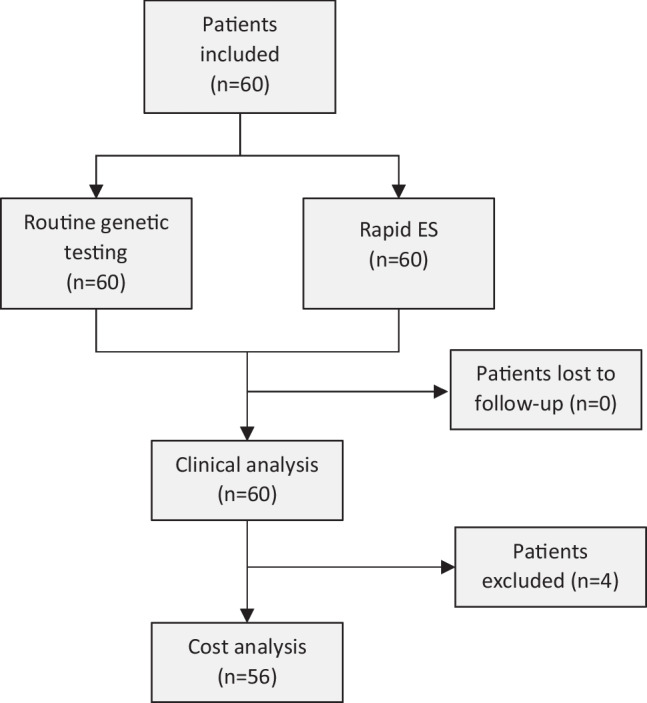
Flowchart of the included patients. Overview of patients enrolled in this study, including patients lost to follow-up or excluded

We performed a multicenter prospective parallel cohort study, in which we assessed the clinical utility of rES compared to conventional genetic testing, i.e., routine genetic testing/the genetic trajectory based on decisions of the clinicians. In order to compare these two genetic trajectories, all study participants received both conventional genetic testing and rES in parallel (Fig. 1). This study design, in which the participants served as their own control, allowed the eliminate potential biases and confounders. Moreover, this approach allowed for direct comparison of both trajectories at three defined outcome measures including (i) genetic diagnostic yield, defined as the percentage of neonates receiving a conclusive genetic diagnosis; (ii) time to diagnosis (TTD), calculated as the time between the request of the first genetic test and receiving the conclusive genetic test results; and (iii) costs associated with (genetic) healthcare resource use in the first 2 years of life.

This study was approved by the Medical Research Ethics Committee Arnhem/Nijmegen under file number 2016–2486/ NL57511.091.16.

### Patient recruitment

We studied 60 neonates admitted to a NICU in five out of ten centers in the Netherlands between May 2017 and January 2019. This sample size was calculated based on a two-sided chi-square test, using a power of 80% and a significance level of 0.05. The diagnostic yield of routine genetic testing was estimated to be 5% and 25% for rES. The sample size calculating resulted in a minimum of 55 patients, with a significance level of < 0.001. Taking into account a possible drop out of 10%, during the course of the study, 60 patients were recruited. Criteria for inclusion were a postnatal age less than 3 months at presentation and high suspicion of a genetic disorder (as assessed by neonatologist and/or clinical geneticist based on the neonate’s clinical presentation). In addition, EDTA blood samples of both biological parents were required for participation. Exclusion criteria were a previously (prenatally) established genetic diagnosis or a clinically phenotype highly associated with trisomy 13, 18, 21, or monosomy X. Parents were informed about the study by the attending clinician, in consultation with a clinical geneticist, if routine genetic testing was indicated. Written informed consent was obtained for all participating families.

### Clinical description to assess representativeness of cohort

Clinical features were scored using the human phenotype ontology (HPO) terms [15] (Supplementary Table 1). CA were considered isolated when affecting a single organ system and as multiple congenital anomalies (MCA) when affecting two or more organ systems. Representativeness of the cohort was determined by comparison to our recent analysis of a retrospective cohort of > 1400 neonates admitted to the NICU for which we described routine genetic care, uptake of genetic testing, and diagnostic potential, in addition to economic models predicting effects of the use of rES in a NICU setting [14, 16].

### rES procedure

The rES procedure was performed in the ISO15189 accredited genetic diagnostic laboratories affiliated to the five NICUs. Whereas minor technical differences exist between centers, such as, for instance, different enrichment kits or sequencing equipment used, the overall procedures were similar [17–19]. Importantly, data interpretation was in all neonates guided by the clinical referral and could consist of interpretation of disease gene-specific panels, interpretation of the Mendeliome (all genes with confirmed OMIM–disease–gene associations), interpretation of all genetic variants to allow discovery of novel candidate disease genes (open exome strategy), or a combination of these strategies. Variants were clinically interpreted based on a 5-class system, with class 1/2 representing (likely) benign variants, class 4/5 representing (likely) pathogenic variants, and class 3 representing variants of unknown clinical significance [20].

### Diagnostic yield

For each neonate, we monitored the routine genetic diagnostic trajectory (Supplementary Table 2). In routine care, the type of genetic tests and the number of tests were left at the discretion of the clinical geneticist. In parallel, rES was performed as study intervention. For neonates where ES was requested as part of routine diagnostic care, the ES was not performed in duplicate, but the results of the rES were used as such. A conclusive diagnosis was defined as a laboratory-confirmed genetic diagnosis based on the identification of a (likely) pathogenic (class 4 and 5) variant in concordance with the patients’ phenotype. Variants of unknown clinical significance (class 3) in a known disease gene in concordance with the neonates’ phenotype were considered a possible diagnosis. For comparison of the diagnostic yield, only conclusive diagnoses were considered.

### Turnaround time and time to diagnosis

To gain insight into the time spent to obtain a genetic diagnosis, we discriminated between the TAT and the TTD. The TAT could be assessed for all tests and describes the time between receipt of the diagnostic sample and the final test report, irrespective of the obtained result (e.g., diagnosis or no diagnosis). In contrast, the TTD was only determined for neonates who received a conclusive diagnosis. The latter was chosen as this reflects an objective end point of genetic diagnostic care for index patients. The TTD was measured from the moment the first genetic test was requested by the involved clinician until the return of the final conclusive genetic diagnostic report. Of note, in case ES was request in the conventional diagnostic strategy, only the rES was performed, and the TAT of routine ES was censored to overcome the need to perform the ES procedure twice. To determine this censored TAT of ES in conventional diagnostic testing, the TAT of ES was determined from a random, anonymized, set of individuals, unrelated to this project, but equal in size of the number of neonates receiving rES. This resulted in a TAT for ES of 105 days (95% CI 96–113 days).

### Costs of genetic diagnostic trajectory

Healthcare consumption was collected from the electronic patient file of 56/60 patients. For four neonates, these data could not be collected, and therefore, these patients were excluded from the cost analyses. Healthcare activities were linked to their unit prices (index year 2020) retrieved from the Dutch Healthcare Authority (Nederlandse Zorgautoriteit (NZA)) and the National Healthcare Institute (Zorginstituut Nederland (ZiN)) [21, 22], after which a distinction was made between costs related to genetic diagnostic testing and other healthcare-related costs.

For all healthcare data, average costs per neonate were calculated, including the minimum, maximum, and median. Costs were divided into seven categories based on their type of healthcare activity: diagnostics, hospitalization, consult, surgery, medicines, genetic, and other healthcare costs. Per category, a percentage of total healthcare costs was calculated. For the category “genetic costs,” the types of genetic tests performed were also retrieved to provide more detailed insight to the build-up of costs associated with genetic testing.

Next, two cost analyses were performed, firstly the economic impact of implementation of rES compared to the conventional genetic testing, and secondly, the evaluation of the timing of expenditure for genetic testing, differentiating between costs made during the neonatal period (first 28 days starting at birth) and costs during the post-neonatal period with a maximum follow-up period of 2 years after birth.

### Statistical methods

Data analysis of the clinical variables was performed in Excel (version 2016), and the cost analysis was performed in R (version 4.0.3) [23]. In more detail, normal distributed data were expressed in mean and standard deviation; median and interquartile ranges were used in data with a skewed distribution. Paired samples *t* test was performed to analyze whether the difference in TAT and costs between the two diagnostic trajectories were significant.

## Results

### Representativeness of cohort

A total of 60 newborns admitted to the NICU with clinical features suggestive for a possible genetic disorder were enrolled in this study. Demographic information is presented in Table 1, with detailed clinical characteristics per patient summarized in Supplementary Table 1. When comparing the cohort to a recently published, retrospectively collected cohort of NICU patients [14], we observe a shift towards more newborns with CA (78% vs. 32%, *p* < 0.001), as expected given our study purpose and the relation between genetic disorders and CA [14]. Patients with CA are more frequently genetic tested than neonates without. Within the subgroup of neonates with CA, neonates more often showed multiple CA (70%) than isolated anomalies (30%).

**Table 1:** Clinical characteristics

	*N* = *60*
Male/female	32 (53%)/28 (47%)
*Gestational age*	
Extremely preterm (< 28 weeks)	7 (12%)
Very preterm (28 weeks to 33 weeks)	15 (25%)
Preterm (34 weeks to 36 weeks)	8 (13%)
Term (37 weeks to 41 weeks)	29 (48%)
Post-term (> 42 weeks)	1 (2%)
*Clinical features*	
Congenital anomalies	47 (78%)
Isolated	14/47 (25%)
Multiple	33/47 (53%)
No congenital anomaly	13 (22%)
*Prenatal*	
Prenatal ultrasound abnormalities	30 (50%)

### Comparison of diagnostic yield, TAT, and TTD in routine genetic testing versus rES

For all 60 neonates, the genetic diagnostic trajectory started in the neonatal period, at an average age of 8 days (95% CI 6–10 days). From this start point onwards, the neonates received both routine genetic testing and rES in parallel, allowing to directly compare outcome measures as neonates served as their own controls.

In routine genetic testing, a total of 112 genetic tests were performed, resulting in an average of 1.87 tests per patient (range 1–5; Supplementary Fig. 1; Supplementary Table 2). In total, 7 different types of assays were requested, with genomic microarray (46/112; 41%) and routine ES (44/112; 39%) being most frequently ordered. In 6 out of 60 neonates (10%), a conclusive genetic diagnosis could be identified (Table 2). The mean TAT of the routine genetic diagnostic trajectory by tests initiated in the neonatal period was 81 days (95% CI 71–92), with the main driver of this relatively long TAT being routine ES (Supplementary Table 2). For patients with a conclusive diagnosis, the average TTD was 59 days (95% CI 23–98).

**Table 2:** Genetically confirmed diagnosis and diagnostic methods

		**Rapid ES**		
		**Conclusive diagnosis**	**No diagnosis**	**Total**
**Conventional genetic testing**	**Conclusive diagnosis**	**6** - 22q11 deletion syndrome (2 ×) - Mowat–Wilson syndrome - Renal cysts and diabetes syndrome - Trisomy 21 - Turner syndrome^a^	**0**	**6**
	**No diagnosis**	**6** - Costello syndrome - Developmental and epileptic encephalopathy - Noonan syndrome (3 ×) - X-linked myotubular myopathy	**48** *Undiagnosed patients*	**54**
	**Total**	**12**	**48**	

*ES* exome sequencing

^a^in this case, rES detected a possible 45,X0 based on variant characteristics on the X-chromosome, the conventional genetic test, performed in parallel to rES, confirmed a 45,X0 chromosome profile

Parallel to the conventional genetic diagnostic trajectory, all neonates received trio-based rES. These efforts resulted in conclusive genetic diagnosis in 12 of 60 neonates (20%), providing higher diagnostic yield than the routine genetic tests (Table 2). rES had an average TAT of 12 days (95% CI 10–14 days), being significantly shorter than the average 81-day TAT in routine genetic testing (*p* < 0.001; Supplementary Table 2). The average TTD was 15 days (95% CI 10–20) for patients with a conclusive genetic diagnosis, which is four times faster than for routine genetic testing (59 days).

### Concordance between genetic diagnostic trajectories

In our cohort of 60 neonates, 12 conclusive genetic diagnoses were obtained (Supplementary Table 3). Eight of the 12 (66%) diagnoses were based on (de novo) single-nucleotide variants (SNVs), known to lead to monogenic disorders with high genetic and clinical heterogeneity. In two neonates (17%), an aneuploidy was detected including trisomy 21 resulting in Down’s syndrome, and 45,X0 causing Turner syndrome without a typical phenotype. The remaining two diagnoses (17%) were based on smaller copy number variants (CNVs). Of these 12 diagnoses, 6 were detected by both genetic diagnostic trajectories, albeit that for one of these, an additional confirmatory test was needed in the rES trajectory. The remaining six diagnoses were only obtained in the genetic trajectory using rES (Table 2).

### Comparison of costs of genetic diagnostic trajectories

In the routine genetic care pathway, the average total healthcare costs during the first 2 years of life were €125,826 per neonate (Table 3), with 5.2% of the expenditures (on average €6568 per neonate) for genetic care. Of the expenditure for genetic care, 83.6% (on average €5494 per neonate) corresponded to routine genetic diagnostic testing, and 16.4% (on average €1074 per neonate) was spent on genetic consultation.

**Table 3:** Average costs per patient

	**Average per patient (€)**	**Min (€)**	**Median (€)**	**Max (€)**	**Percentage**
**Diagnostics**	16,648	234	8310	101,206	13.2%
**Hospitalization**	97,552	9038	53,273	318,838	77.5%
**Consult**	2725	0	1606	14,658	2.2%
**Surgery**	519	0	0	11,045	0.5%
**Genetics**	6568	392	6882	14,964	5.2%
**Medicines**	519	0	0	22,608	0.4%
**Other**	1223	0	179	12,205	1.0%
**Total**	125,826	10,273	69,224	416,473	

**Fig. 2:**
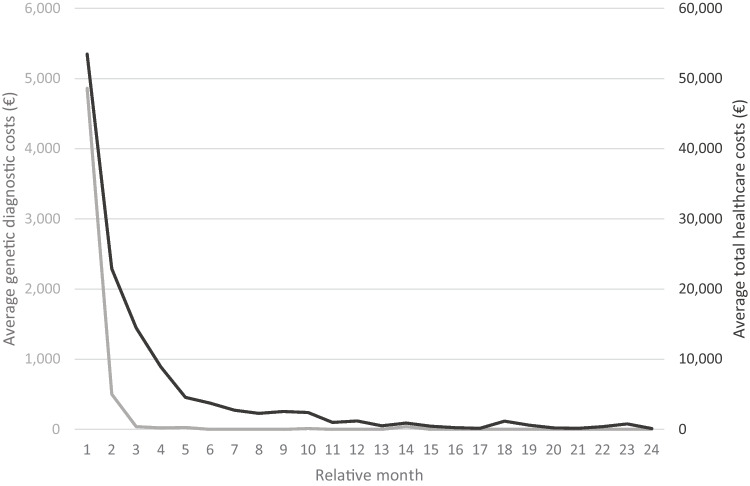
Average genetic costs per month per patient over time. Overview of average genetic diagnostic costs (gray) and average total healthcare costs (black) over time, starting from birth (relative month). Costs decrease over time, and 88% of the average genetic diagnostic costs are made during the neonatal period

To assess the overall impact rES as replacement for the routine genetic testing, costs of routine genetic testing (€5494 per neonate) were substituted by the costs of trio-based rES (€5409 per neonate; Table 4), resulting in a (non-significant, *p* = 0.23) reduction in genetic costs by 1.5% (i.e., €85 per neonate). We subsequently aimed on gaining insight into the timing of healthcare utilization and associated costs (Fig. 2). Of the €5494 for genetic diagnostic testing, €4781 (87%) was spent in the neonatal period (Fig. 2).

**Table 4:** Average number of declarations and associated costs per neonate

		**Average number of declarations per neonate**	
**Genetic diagnostic test**	**Unit price (€)**	**Routine genetic testing**^**c**^	**Rapid ES**
Karyotype	933	0.23	-
FISH	818	0.05	-
Genomic microarray	825	1.09	-
Sanger sequencing	561	0.13	-
Disease specific gene	871	0.05	-
Gene panel	1,768	0.04	-
ES^a^	5,409	0.73	1.00
Other^b^	640	0.30	-
**Total**		**2.62 (€5,494)**	**1.00 (€5,409)**

*FISH* fluorescence in situ hybridization, *ES* exome sequencing

^a^Trio-ES, so the unit price of singleton ES (€1,803) was multiplied by three, ^b^follow-up genetic test in one or more genes, ^c^of note, the number of average tests performed in routine care on the index case (i.e., 1.87) deviates from the average number of total declarations as also parental samples are tested and results in healthcare-related costs to obtained diagnosis

## Discussion

We studied the genetic diagnostic trajectory of ill newborns with suspected genetic disease to evaluate the clinical utility of rES in comparison with conventional genetic diagnostic testing in a multicenter prospective observational study at tertiary NICUs throughout the Netherlands. We demonstrate that the use of rES in neonates with a suspected genetic disorder is associated with a higher diagnostic yield and leads to a faster diagnosis, without an increase in costs.

This study showed that rES had a diagnostic yield of 20%. This diagnostic rate was lower compared to other studies yielding 36–57% in neonatal settings [24, 25]. This might be explained by our clinical preselection that was aimed to represent all neonates admitted with a disorder suspected of a genetic origin. These inclusion criteria in this study were less strict compared to previous studies. Besides, the makeup of the group of newborns for this study can be influenced by the difference in genetic expertise of the clinicians involved in the selection, namely, pediatricians and not clinical geneticists. In addition, we excluded neonates who already received prenatal positive rES testing. With prenatal rES having found its place in routine testing upon ultrasound abnormalities in the same time as when this clinical utility study was performed, it is also within reason to expect that this has influenced the overall diagnostic yield in this cohort [17]. Nonetheless, given the broad spectrum of diseases studied, rES has been shown to be a suitable genetic diagnostic tool for neonates suspected of a genetic disorder in different disease areas. Although some genetic diseases exhibit themselves within the first 28 days of life or shortly thereafter, some clinical symptoms may be undifferentiated, especially in the early days of life [26]. We found that the presence of MCA increased the likelihood of finding a conclusive genetic diagnosis.

Identifying the genetic cause of disease may lead to patient tailored clinical management and facilitate shared decision-making regarding end-of-life decisions, either way reducing ineffective, empirical, or detrimental therapies [27]. As an example, in one of the included patients with a severe congenital heart defect, epilepsy, and agenesis of corpus callosum, the diagnosis Mowat–Wilson syndrome was established. Importantly, a former study of our group also demonstrates a high diagnostic rate for MCA [14]. This diagnosis was only identified by the rES trajectory and helped the parents and the involved clinician in their decision about optimal care. This prevented ineffective cardiorespiratory support given the severe prognosis related to the underlying genetic disorder and withdrawal of intensive treatment was initiated.

Based on our recent study in which we modeled scenarios for the implementation of rES for neonates admitted to the NICU based on a retrospective cohort of > 1400 neonates, it was expected that the use of rES would lead to an increase of genetic diagnostic costs [14, 16]. However, establishing a genetic diagnosis early in life would also prevent a diagnostic odyssey and was considered the main incentive, along with potential future saving on futile testing, for the use of rES in critically ill neonates admitted at the NICU [27, 28]. Our prospective evaluation however shows that, in addition to an increased diagnostic yield and a shorter TTD, rES leads to reduced costs related to genetic testing. A likely explanation for this difference in anticipated increase, and observed decrease in costs, is identified by the uptake of ES as part of the routine genetic diagnostic trajectory (e.g., with an average TAT of 81 days). Results of this study showed that ES is implemented during the routine genetic diagnostic trajectory, which caused a larger increase in costs compared to the genetic trajectory in which only rES was performed. In our retrospective analysis, ES was only rarely used. Prospectively, we now noticed that ES was requested in 73% (44/60) of the cohort, despite the realization of neonatologists and clinical geneticist that the TAT would be too long to impact decision-making while at the NICU; the prevention of a diagnostic odyssey prevailed. Our study now objectively shows that rES, as such, is not increasing costs compared to the current use of genetic diagnostics and highlights that systematic use of rES for all neonates with (M)CA, admitted at the NICU, would increase diagnostic yield. Interestingly, our longitudinal data of 2-year follow-up showed that there is also limited uptake of additional genetic testing after the initial rES, and > 95% of genetic diagnostic costs concentrated in the neonatal period. However, costs associated with (periodic) re-analysis of the existing rES data for neonates without a genetic diagnosis are still to be expected. To what extent these additional costs outweigh costs saving elsewhere in the care path of these patients is still to be determined. However, based on earlier research, it is assumed that shortening of the diagnostic trajectory by implementing rES will lead to a reduction of healthcare costs, also taking into account cost savings in the future [29–31]. Moreover, withdrawal of care after confirmation of a genetic diagnosis associated with a very poor prognosis will prevent superfluous medical expenses.

Proper genetic consultation of parents of newborns is essential for well-informed decision-making and to prevent harm or decisional regret afterwards [32]. Prior to the start of our study, several patient associations were hesitant about implementing rES in neonates admitted to the NICU because of the potential extra (emotional) burden it may bring to parents of neonates. We performed an explorative study about the (emotional) burden of parents and clinicians (neonatologists), showing that most of the parents experienced use of rES not as stressful and evaluated it as having a positive impact on clinical decision-making (Supplementary Fig. 2). Dedicated studies with validated questionnaires are, however, needed to substantiate firm conclusions in end-user perspectives.

A limitation of our study is the relatively small sample size per center, hampering the analysis on representativeness of the cases per center. However, our approach did allow us to more broadly assess feasibility of rES implementation since multiple centers participated in this study. Furthermore, there is no significant difference between the included centers regarding clinical decision-making. Therefore, we expect no limitations regarding generalizability of the results. As these NICUs are also referral centers with a high volume of transfers and retro-transfers, it is difficult to capture an infant’s entire diagnostic odyssey, particularly if it began in another institution. However, our study is valuable in reflecting current practices at NICUs that cares for many neonates with rare and likely genetic disorders.

Overall, it can be concluded that rES as a first-tier genetic test in the NICU is clinically relevant and beneficial for newborns, their parents, and treating clinicians. Implementation of rES will increase diagnostic yield and provides a diagnosis more rapidly than conventional genetic testing, without incurring higher costs to the healthcare system enabling individualized clinical management.

## Supplementary Information

Below is the link to the electronic supplementary material.

Supplementary file1 (DOCX 74 KB)

Supplementary file2 (XLSX 19 KB)

Supplementary file3 (XLSX 16 KB)

Supplementary file4 (XLSX 16 KB)

## Abbreviations

NICU: Neonatal intensive care unit
(M)CA: (Multiple) congenital anomalies
rES: Rapid exome sequencing
TAT: Turnaround time
TTD: Time to diagnosis
