# Supplementary material for: Rapid exome sequencing as a first-tier test in neonates with suspected genetic disorder: results of a prospective multicenter clinical utility study in the Netherlands
Source: Eur J Pediatr. 2023 Mar 31;182(6):2683–92. doi: 10.1007/s00431-023-04909-1 (PMC10257607; doi:10.1007/s00431-023-04909-1)
Supplement: Supplementary file 1 — Supplementary file1 (DOCX 74 KB) [file 431_2023_4909_MOESM1_ESM.docx]

**Supplementary figure 1 Overview of all genetic test**

ES; exome sequencing; *Contains quantitative fluorescent polymerase chain reaction (QF-PCR) and fluorescence in situ hybridization (FISH)

**Supplementary figure 2 End user perspectives.**

**
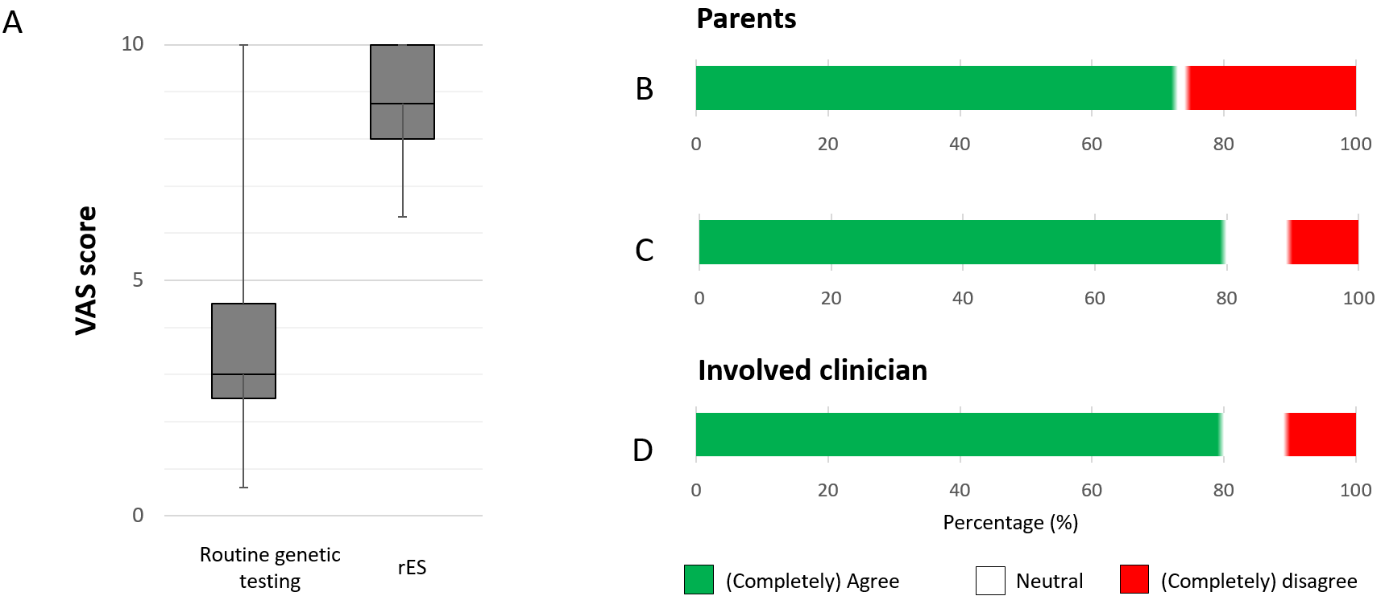
**

Exploratory analysis to gain insight into the perspectives of parents of neonates receiving rES as well as the involved clinicians (i.e. neonatologist). (**A**) The parental perspective was focused on their overall experience of the rES procedure compared to routine testing. Overall experience was assessed by the visual analog scale (VAS), a 10-point scale ranging from 0 (extremely unsatisfied) to 10 (extremely satisfied) in which parents answered the following questions: 1) “*To what extend are you satisfied about the rapid genetic diagnostic testing?*”; and 2) “*How satisfied would you be if genetic test results are available within 3-4 months (i.e. routine genetic diagnostic testing)?*”. In addition, to assess the perceived stress related to rES, based on the differences in procedure such as the risk of incidental findings associated with rES, parents indicated whether they agreed with the following two statements by using a 7-point scale ranging from completely agree to completely disagree: “*It did not cause stress in order to decide whether rES should be performed.*” (**B**) ; and 2) “*If we had the opportunity to choose again for this diagnostic trajectory, we would choose again for rapid genetic diagnostic testing.*” (**C**). The involved clinicians’ perspective was focused on the speed of rES to have an impact on clinical decision making (7-point scale, ranging from completely agree to completely disagree). They indicated to what extend they agreed with: “*The TAT of rES is short enough to impact clinical decision making.*” (**D**). These exploratory questions and answer suggest that i) parents favoured the rES over routine testing, ii) they experienced no extra stress related to testing and iii) that they would again choose rES if they had the opportunity. From the involved clinician’s point-of-view, it seemed that the results from rES were fast enough to impact clinical decision making.
